# Alignment Between Heart Rate Variability From Fitness Trackers and Perceived Stress: Perspectives From a Large-Scale In Situ Longitudinal Study of Information Workers

**DOI:** 10.2196/33754

**Published:** 2022-08-04

**Authors:** Gonzalo J Martinez, Ted Grover, Stephen M Mattingly, Gloria Mark, Sidney D’Mello, Talayeh Aledavood, Fatema Akbar, Pablo Robles-Granda, Aaron Striegel

**Affiliations:** 1 Computer Science and Engineering University of Notre Dame Notre Dame, IN United States; 2 Informatics Department University of California Irvine, CA United States; 3 Institute of Cognitive Science University of Colorado Boulder Boulder, CO United States; 4 Department of Computer Science Aalto University Espoo Finland; 5 Thomas M Siebel Center for Computer Science University of Illinois Urbana-Champaign Urbana, IL United States

**Keywords:** stress measurement, heart rate variability, HRV, perceived stress, ecological momentary assessment, EMA, wearables, fitness tracker

## Abstract

**Background:**

Stress can have adverse effects on health and well-being. Informed by laboratory findings that heart rate variability (HRV) decreases in response to an induced stress response, recent efforts to monitor perceived stress in the wild have focused on HRV measured using wearable devices. However, it is not clear that the well-established association between perceived stress and HRV replicates in naturalistic settings without explicit stress inductions and research-grade sensors.

**Objective:**

This study aims to quantify the strength of the associations between HRV and perceived daily stress using wearable devices in real-world settings.

**Methods:**

In the main study, 657 participants wore a fitness tracker and completed 14,695 ecological momentary assessments (EMAs) assessing perceived stress, anxiety, positive affect, and negative affect across 8 weeks. In the follow-up study, approximately a year later, 49.8% (327/657) of the same participants wore the same fitness tracker and completed 1373 EMAs assessing perceived stress at the most stressful time of the day over a 1-week period. We used mixed-effects generalized linear models to predict EMA responses from HRV features calculated over varying time windows from 5 minutes to 24 hours.

**Results:**

Across all time windows, the models explained an average of 1% (SD 0.5%; marginal *R*^2^) of the variance. Models using HRV features computed from an 8 AM to 6 PM time window (namely work hours) outperformed other time windows using HRV features calculated closer to the survey response time but still explained a small amount (2.2%) of the variance. HRV features that were associated with perceived stress were the low frequency to high frequency ratio, very low frequency power, triangular index, and SD of the averages of normal-to-normal intervals. In addition, we found that although HRV was also predictive of other related measures, namely, anxiety, negative affect, and positive affect, it was a significant predictor of stress after controlling for these other constructs. In the follow-up study, calculating HRV when participants reported their most stressful time of the day was less predictive and provided a worse fit (*R*^2^=0.022) than the work hours time window (*R*^2^=0.032).

**Conclusions:**

A significant but small relationship between perceived stress and HRV was found. Thus, although HRV is associated with perceived stress in laboratory settings, the strength of that association diminishes in real-life settings. HRV might be more reflective of perceived stress in the presence of specific and isolated stressors and research-grade sensing. Relying on wearable-derived HRV alone might not be sufficient to detect stress in naturalistic settings and should not be considered a proxy for perceived stress but rather a component of a complex phenomenon.

## Introduction

### Motivation and Overview

The World Health Organization classified stress as a 21st-century epidemic [1], as chronic stress can have adverse effects on health and well-being. Stress is the perceived imbalance in demands and resources and is experienced when a situation is appraised as personally significant and taxes or exceeds resources for coping [2]. In the short term, stress is associated with negative feelings, decreased performance and productivity, and muscular problems such as tension and headaches [3,4]. In the long term, stress can lead to significant health problems, including cardiovascular disease, impaired immunity functions, and lower overall quality of life [5,6]. Therefore, the ability to monitor stress through unobtrusive means could help improve health outcomes and well-being.

Stress measurements fall roughly into two broad categories: measuring stress directly through physiological markers such as heart rate (HR) variability (HRV) [7,8], cortisol [9], or electrodermal activity [10] and using physiological data to predict perceived stress using self-reports as ground truth [11-14]. Theories on the role of appraisal on the stress response suggest a positive relationship between perceived stress (through appraising a situation as threatening or demanding) and physiological reactions such as changes in cortisol (ie, the stress hormone), respiration, and HR [2,15-17]. Laboratory studies generally confirm this relationship (see the *Background* section). However, measuring perceived stress in daily life remains an exceedingly challenging task.

Gold standard biological measures of stress such as cortisol (a stress hormone) tend to be time consuming, expensive, and intrusive; they do not allow continuous measurement and may not align with self-reports [18,19]. Researchers have considered other physiological measures associated with the stress response such as HRV, electrodermal activity, and respiration, which can be obtained using less intrusive means such as wearable sensors [20-22]. Wearable sensors are some of the least intrusive methods of measuring physiological stress and yield continuous measures with increased frequency and finer temporal granularity than self-reports or cortisol samples. In recent years, the increased quality and battery life and the low cost of wrist-worn wearables have made it possible for studies to focus on the alignment between physiological (HRV) and self-reported measures in daily life [12,23,24], bringing to light some of the limitations of translating laboratory findings to real-world settings.

Although laboratory studies that induced stress supported an association between HRV and perceived stress (eg, using the Stroop Color-Word Interference Test and mental arithmetic problems [25-28]; also see the study by Kim et al [29] for a review that found differences in HRV in response to stress), studies in daily life settings with and without wearables have yielded mixed results. For instance, in a study of 223 male white-collar workers, Kageyama et al [30] found that daily job stressors did not correlate with short-term electrocardiogram (ECG)–derived HRV features. In contrast, in a study of 909 participants, Sin et al [31] found that ECG-derived HRV features negatively correlated with longer-term (as opposed to daily) perceived stress measured over a period of 8 days. Similarly, Hynynen et al [32] found that HRV measured in an orthostatic test (sitting up after a period of sleep) but not during night sleep was related to longer-term self-reported (global) stress over the past month. Specifically, HRV features were lower in the group with high stress than in the group with lower stress, whereas HR was higher in the group with high stress. Furthermore, in a study of 20 surgeons monitored continuously over 24 hours, Rieger et al [33] separated surgeons into groups experiencing high and low stress and found significantly higher HR and lower HRV during sleep in the group with high stress.

In real-world settings involving wearables, few studies have used HRV to predict perceived stress and have also found mixed results. Hernandez [23] collected physiological and behavioral data to predict self-reported momentary stress (high vs low) from 15 participants during 5 regular days of work.

Hernandez [23] used a support vector machine model using HRV features, achieving an average accuracy of 56%, slightly better than the 50% at baseline. Similarly, in a 4-month study of 35 participants, Muaremi et al [12] achieved a classification accuracy of 59% in a 3-level prediction task of perceived stress (low, moderate, and high), with 40% at baseline. In a simpler classification task of high versus low stress, Wu et al [24] found that HRV features yielded a classification accuracy of 78% in a study of 8 participants for 2 weeks in a data set with 59% of the samples corresponding to low stress.

These studies demonstrate that HRV associations with perceived stress obtained in situ and with wearables are less consistent than in laboratory studies. The evidence is inconclusive as to whether HRV in real-life settings could reflect *daily* or *momentary* perceived stress, as is often assumed in popular applications [8,34-37]. The greatest success comes from a few small-scale studies with simplified (eg, binarized from ordinal ratings with the removal of the more difficult middle cases) stress classification tasks. Given the recency of incorporating HRV measurement in consumer-grade wearable devices to track stress in daily life and the lack of large-scale studies addressing this issue, we report on a main study, where we collected HRV data from wrist-worn wearables, as well as self-reports for 657 participants across 9 weeks, and a follow-up with 327 (49.8%) of the same participants over 1 week approximately a year later.

We extend previous studies that predicted stress from wearable HRV data in two ways: (1) we collected HRV data in a large-scale longitudinal study in a naturalistic setting (ie, without control over what stressors occur and when); and (2) we incorporated retrospective stress evaluations, including measures of the timing of stressful periods, to investigate whether contextual knowledge of when stress occurs could help predict perceived stress. Our studies also aimed to shed light on potential factors that could explain why self-reports of stress often do not correlate with physiological measures. Specifically, we aimed to understand the extent to which HRV predicts perceived stress in naturalistic settings. Furthermore, given that HRV is a measure of arousal, we also examined the extent to which HRV is specific to stress beyond other high-arousal affective states, including anxiety, negative affect, and positive affect.

The contributions of this study are as follows:

1. We quantified the degree of association between HRV and perceived stress in a longitudinal large-scale in situ study with information workers.
2. HRV can be calculated in many ways over many time scales (eg, 5 minutes to 24 hours). We identified low frequency (LF)/high frequency (HF) ratio, very LF (VLF), triangular index, and SD of the averages of normal-to-normal intervals (SDANN) calculated between 8 AM to 6 PM as the HRV features most strongly associated with perceived stress. Using these optimal features, we found that HRV is a predictor of perceived stress; however, the relationship is not as strong as in the laboratory, indicating that HRV is limited as a sole indicator of perceived stress, as is often used in modern applications.
3. We found that the same features that indicate stress also predict anxiety, negative affect, and positive affect. However, HRV still uniquely predicts stress after accounting for the shared variance of these related constructs with stress.
4. We describe the limitations of using HRV to measure perceived stress in situ and offer suggestions to improve perceived stress measurement.

### Background

Stress is defined as the physiological response to maintain homeostasis in unexpected situations or when perceiving a threat [38-41]. The stress response is manifested in 2 systems, the autonomic nervous system (ANS)—through the sympathetic nervous system (SNS) and parasympathetic nervous system (PNS)—and the hypothalamic-pituitary-adrenal (HPA) axis [42]. The SNS outputs epinephrine, which promotes rapid and widespread physiological changes such as increased HR [43,44], whereas the PNS generally does the opposite [40,45-47]. The HPA axis outputs cortisol, a stress hormone, which supports the SNS system by increasing available glucose by suppressing other body systems such as immune function and growth [5,48,49]. In general, SNS activity ends when a stressor ends, whereas HPA axis activity may persist for up to 90 minutes after the stressor ends [50-52]. Thus, especially over time and with chronic stressors (eg, caregivers of patients with dementia), there may be a sustained cortisol response in the absence of specific SNS activity [53-55]. Many of the chronic detrimental effects of stress, such as the increased risk of heart disease, diabetes, and mortality, are associated with increased cortisol [5,56-58].

HRV is a measure of ANS activity and has been associated with health and physical and mental stress [25,29,59-65]. HRV measurement relies on the detection of RR intervals; that is, the time between upward deflections in an ECG. Effective clinical ECG measurements require the assistance of a trained clinician to ensure correct electrode placement. A more user-friendly version for (fitness conscious) consumers is chest straps (eg, Zephyr Bioharness [66,67]) that capture waveforms in the same manner as an ECG and do not require a clinician while still being vulnerable to improper positioning.

At the other end of the spectrum, photoplethysmography sensors approximate the measurement of RR intervals by detecting beat-to-beat intervals (BBI) evidenced by volumetric changes in the microvascular bed of tissue [68,69]. Traditionally used in wearable equipment such as fitness trackers, smartwatches, and armbands, they are easy to fit and have extended battery life, therefore allowing for continuous measurement of BBI and, in consequence, HRV. This has enabled a myriad of applications that use these sensors to measure HRV and provide a measurement of “stress” [8,34-37]. However, although HRV is associated with stress in laboratory studies, as discussed previously, HRV only measures one component of the stress response: ANS activity. Although the short duration and acute stressors may evoke a strong SNS response, chronic stressors that are characterized by increased cortisol in the absence of an SNS response may not be detected by HRV alone but could still influence self-reports of perceived stress.

The differences between SNS and HPA axis activity, their measurement, and the time courses of responses may play a role in when (or whether) a relationship is found between physiological responses and self-reported stress (eg, cortisol assessed via blood shows faster responses than cortisol measured by saliva). For instance, one study [51] induced stress and found that self-reported stress was associated with physiological stress (increased HR and cortisol) only if assessed during the stressor task. Self-reported stress before or after the stressor did not correlate with physiological stress during the same period. Other studies suggest there may be a lag between perceived and physiological stress where subjective stress responses precede cortisol (endocrine) responses [70]. Gaab et al [71] found that anticipatory but not retrospective cognitive appraisal of stress (self-report) is an important determinant of the cortisol stress response, indicating that the timing of the self-report in relation to the stressor affects whether a relationship is found between perceived and physiological stress. In contrast, Oldehinkel et al [72] found that perceived stress *before* a social stressor in the laboratory did not predict physiological responses, although changes in perceived arousal and unpleasantness were associated with changes in HR, respiratory sinus arrhythmia, and cortisol during the stressor. Furthermore, perceived stress measured *after* the stressor was inversely associated with HR during the stressor.

Regarding field studies, in a literature review on the association between salivary cortisol and self-reported stress, Hjortskov et al [18] reported a lack of sufficient evidence of an association between self-reported mental stress and the cortisol response in field studies. The review suggested that the large diversity in study designs and stress measurements possibly obscured any potential relationship. However, these findings from previous studies on the association between perceived and physiological stress indicate a relationship that may be dependent on the temporal resolution of both measurements.

Taken together, the data suggest that HRV is a reliable measure of perceived stress during stressful tasks in the laboratory. However, reliability can be eroded in naturalistic studies for several reasons. First, ecological momentary assessments (EMAs) for stress may not occur (or be answered) during a stressor, which may reduce the accuracy of physiological signals for predicting self-reported stress. Second, HRV-based measures of stress would require a stressor that evokes an HR or HRV response rather than a chronic stressor that may influence self-reports but not HR (eg, a chronic illness). Third, self-reported stress may be reflecting memory biases or coping responses (eg, see the studies by Redelmeier and Kahneman [73] and Scheier et al [74]). Fourth, there are contradictory results for the best time to measure the physiological response of a self-reported stressor (albeit possibly because of methodological differences), coupled with the lack of precise and complete information on stressors that influence the perceived stress level themselves. Finally, HRV measured from wearable sensors might not be sufficiently reliable and might be too sensitive to noise (eg, motion artifacts), thereby obfuscating any potential relationship [70]. Given these challenges, this study sought to investigate the relationship between HRV measured through wearable sensors and perceived stress in a large sample across an extended period and in situ.

## Methods

### Data Collection

This data were collected as part of the larger Tesserae Project [75]. Most participants came from 4 distinct organizations (denoted by O1, O2, O3, and O4), and others from various organizations (denoted by U). Participants were enrolled both on site and remotely. The characteristics of the participants, sensing streams, and study details of the Tesserae study are described in the study by Mattingly et al [75].

Participants were enrolled between January and July of 2018 for the main study, where psychological and physiological measurements of 657 participants were collected during the first 56 days of study participation. This data were used to analyze associations between HRV and self-reported perceived stress. On the basis of the results from this study, we conducted a 1-week follow-up study with 49.8% (327/657) of the same participants in April 2019 to ascertain whether the link between HRV and perceived stress could be improved by refining the self-reporting procedure.

### Demographics

Demographics were collected from a survey administered at the onset of participation (Table 1).

**Table 1:** Demographics summary for each study (N=657).

Variable		Main study	Follow-up study (n=327)
**Gender, n (%)**			
	Male	391 (59.5)	211 (65.5)
	Female	266 (40.5)	116 (35.5)
**Organization, n (%)**			
	O1^a^	165 (25.1)	109 (33.3)
	O2^a^	237 (36.1)	78 (23.9)
	O3^a^	85 (12.9)	52 (15.9)
	O4^a^	25 (3.8)	5 (1.5)
	U^b^	145 (22.1)	83 (12.6)
**Supervisor status** **, n (%)**			
	Nonsupervisors	370 (56.3)	206 (63)
	Supervisors	285 (43.4)	121 (37)
	Unknown	2 (0.3)	0 (0)
**Age (years)**			
	Values, minimum	20	20
	Values, maximum	68	68
	Values, mean (SD)	35.2 (9.9)	35.9 (10.3)

^a^Distinct organization.

^b^Other organizations.

### Psychological Measures

#### Main Study

**Figure 1:**
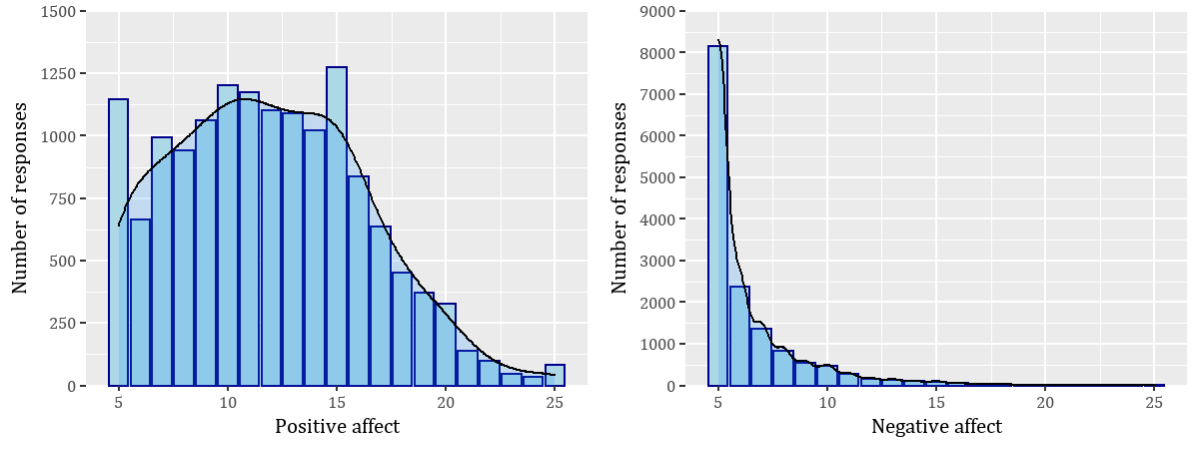
Distribution of positive and negative affect in the main study.

Stress was measured using the question, “Overall, how would you rate your current level of stress?” on a 5-point Likert scale ranging from 1 (no stress at all) to 5 (a great deal of stress); The responses were distributed as follows: 5303 responses were 1s (no stress at all); 5108 responses were 2s (very little stress); 3593 responses were 3s (some stress), 573 responses were 4s (a lot of stress); and 118 were 5s (a great deal of stress). This item was validated in an unpublished study [76] (available upon request) with 991 Mechanical Turk participants (Table S10 in Multimedia Appendix 1 provides correlations with other measures). Affect was measured using the 10-item Positive and Negative Affect Short inventory [77,78]. The distribution of the responses is available in Figure 1. Anxiety was measured using a validated single-item omnibus measure of anxiety, “Please select the response that shows how anxious you feel at the moment,” on a 5-point Likert scale ranging from 1 (not at all anxious) to 5 (extremely anxious) [79]. EMAs were administered once a day through Qualtrics Surveys at 8 AM, 12 PM, or 4 PM over 8 weeks. Participants were prompted to answer the EMAs through SMS text messages. The responses were distributed as follows: 7501 responses were 1s (not at all anxious); 5081 responses were 2s (a little anxious); 1659 were 3s (moderately anxious); 354 were 4s (very anxious); and 100 were 5s (extremely anxious).

Given that the variables were measured repeatedly for each participant throughout the study, we used the repeated-measures correlations [80] procedure to correlate the response variables in the main study. The correlations are shown in Table 2.

**Table 2:** Repeated-measures correlation between response measures in the main study and 95% CI.

Variables	Stress, *r*_rm_ (95% CI)	Anxiety, *r*_rm_ (95% CI)	Negative affect, *r*_rm_ (95% CI)	Positive affect, *r*_rm_ (95% CI)
Stress	1	0.64 (0.63 to 0.65)	0.56 (0.54 to 0.57)	−0.03 (−0.04 to −0.01)
Anxiety	0.64 (0.63to 0.65)	1	0.62 (0.61 to 0.63)	−0.02 (−0.03 to 0.00)
Negative affect	0.56 (0.54 to 0.57)	0.62 (0.61 to 0.63)	1	−0.05 (−0.07 to −0.03)
Positive affect	−0.03 (−0.04 to −0.01)	−0.02 (−0.03 to 0.00)	−0.05 (−0.07 to −0.03)	1

#### Follow-up Study

In the follow-up study, EMAs were sent at 4 PM every day over a week (Monday to Sunday). We collected stress by asking the same item as in the main study along with the following questions: “When did the most stressful part of your day start?”—answered by entering hours and minutes in free-form fields; “When did the most stressful part of your day end?”—also answered by entering hours and minutes in free-form fields; and “How stressful was that time?”—answered on a 5-point Likert scale ranging from 1 (no stress at all) to 5 (a great deal of stress). The responses to the stress question as stated in the main study were distributed as follows: 205 responses were 1s (no stress at all); 530 responses were 2s (very little stress); 484 responses were 3s (some stress), 22 responses were 4s (a lot of stress); and 132 were 5s (a great deal of stress). The responses to the question “How stressful was that time?” were distributed as follows: 36 responses were 1s (no stress at all); 254 responses were 2s (very little stress); 732 responses were 3s (some stress), 71 responses were 4s (a lot of stress); and 280 were 5s (a great deal of stress).

**Figure 2:**
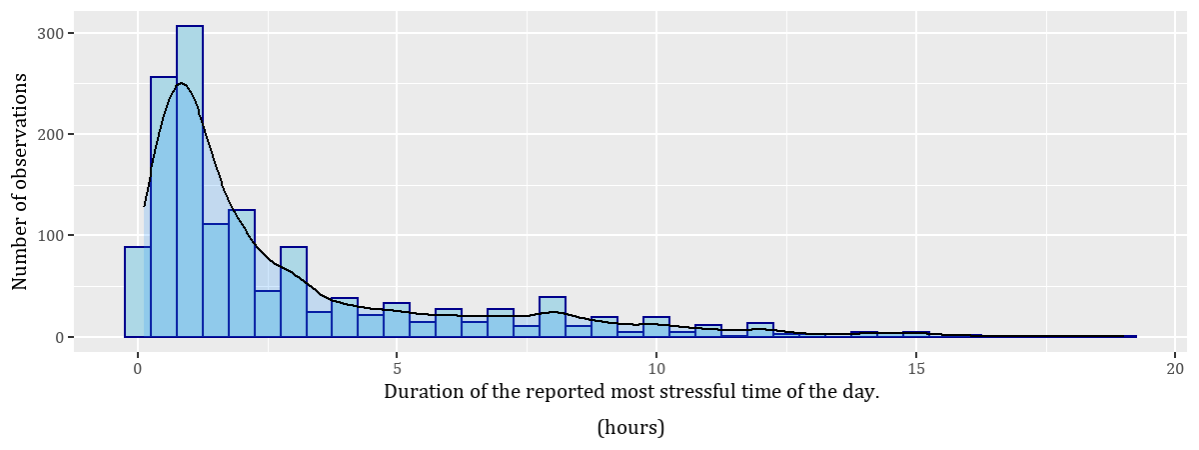
Distribution of the duration of perceived stress at the reported most stressful time of the day. Note that in some cases, this time overlapped with the survey response time.

**Figure 3:**
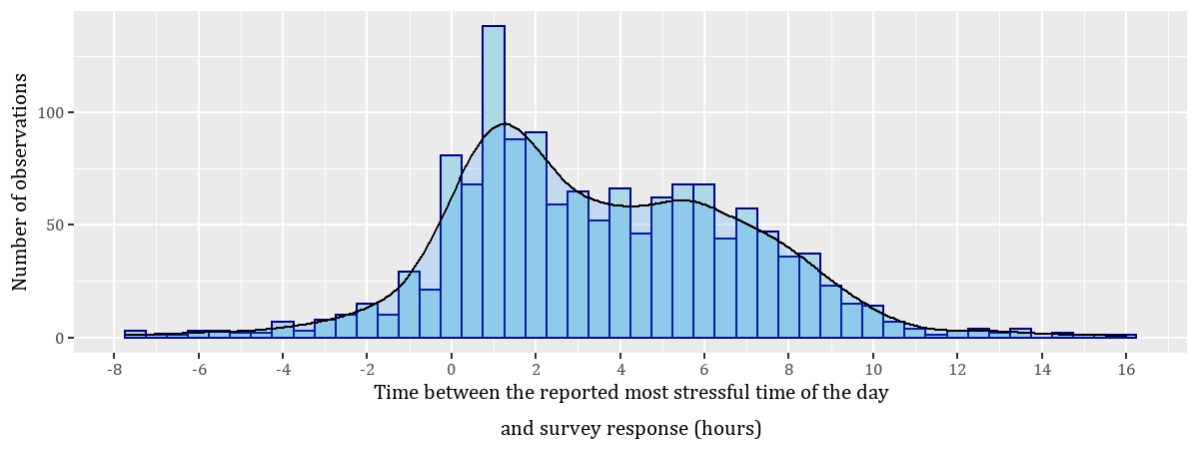
Distribution of the time between the reported most stressful time of the day and the survey response time. Negative values are because of when participants anticipated that the most stressful time of the day would end after the survey response time. Positive times indicate that the most stressful time of the day started and ended before the survey was answered, and negative times indicate the most stressful time of the day at least ended after the survey was answered.

From the timings provided by participants, we calculated the duration of the reported most stressful time of the day, as well as the length of time between the end of that moment and when the participant answered the survey. We refer to the stress question asked in the same way as in the main study, as perceived stress at the time of survey response, whereas we refer to the item introduced in the follow-up study as perceived stress at the reported most stressful time of the day. Figures 2 and 3 provide the distribution of responses, and Table 3 shows the correlation of the responses [80].

**Table 3:** Repeated-measures correlations of the responses in the follow-up study and 95% CI.

Measures	Perceived stress at the time of survey response, *r*_rm_ (95% CI)	Perceived stress at the reported most stressful time of the day, *r*_rm_ (95% CI)	Duration of the most stressful time, *r*_rm_ (95% CI)
Perceived stress at the time of survey response	1	0.5 (0.45 to 0.54)	0.33 (0.27 to 0.38)
Perceived stress at the reported most stressful time of the day	0.5 (0.45 to 0.54)	1	0.17 (0.11 to 0.22)
Duration of most stressful time	0.33 (0.27 to 0.38)	0.17 (0.11 to 0.22)	1
Time between most stressful time and survey response	−0.29 (−0.34 to −0.23)	−0.12 (−0.18 to −0.06)	−0.43 (−0.48 to −0.38)

### Physiological Measures

Wearables can accurately detect HR, especially in conditions of rest or mild exercise [81], although they can have missing data [82]. To measure HR and BBI, from which HRV is computed, participants wore the Garmin vivosmart 3 fitness band (24/7) for the duration of their participation. The same sensors were used in the main study and the follow-up.

In both studies, we examined the associations between HRV and the psychological measures in our sample. To do so, we derived a series of HRV features by adopting standards for the measurement, physiological interpretation, and clinical use of HRV from the North American Society of Pacing and Electrophysiology [29]. In total, we computed 16 HRV features across different time windows using the “hrvanalysis” python library [83], each with a minimum and maximum recording time within the recommended ranges established by Shaffer and Ginsberg [84]. Of these features, 5 were from time domain analyses, which measure variation in HR over time, or the intervals between HR cycles [29]. Triangular index was the single geometric method used [85]. A total of 7 features were from frequency domain analyses [24] where the power spectral density analysis of the HRV frequency domain provides information about how power in a signal is distributed as a function of frequency, which allows the autonomic balance to be quantified at a specific time [29]. The remaining 3 features were nonlinear HRV features, which characterize changes in HRV [86-88]. In this study, we focused on features derived from the Poincaré plot (ie, the scatter plot of successive BBIs: BBI_n_ vs BBI_n+1_). Table 4 shows the mean and SD of the features across 3 different time windows.

**Table 4:** Mean and SD of heart rate variability features in the main study by window size.

Feature	Type	Description	Values by window size, mean (SD)		
			5-minute	8 AM to 6 PM	24-hour
Mean BBI^a^	TD^b^	The mean BBI for a period	758.1 (130.3)	755 (87.1)	797.8 (90.7)
SDNN^c^	TD	The SD of NN^d^ intervals for a period	87.6 (34.2)	135.8 (37.1)	156.0 (45.3)
RMSSD^e^	TD	The square root of the mean of the squares of the successive differences between adjacent NN intervals for a period	68.7 (24.1)	71.7 (18.1)	64.7 (16.1)
PNN50^f^	TD	The number of interval differences of successive NN intervals >50 milliseconds (NN50) divided by the total number of all NN intervals	33.3 (14.4)	33.3 (10.1)	27.8 (9.2)
SDANN^g^	TD	The SD of the averages of NN intervals in all 5-minute segments of a period	N/A^h^	99.5 (32.0)	130.4 (41.4)
Triangular index	GM^i^	The number of total NN intervals/number of NN intervals in the modal bin	16.1 (5.2)	35.4 (10.4)	41.9 (13.5)
HF^j^	FD^k^	Spectral density power in the HF range	1184.9 (688.1)	1244.3 (500.4)	991.0 (404.0)
LF^l^	FD	Spectral density power in the LF range	1637.3 (1129.7)	1779.9 (799.3)	1628.1 (707.0)
LFnu^m^	FD	LF power in normalized units: LF/(total power – VLF^n^) × 100	56.8 (8.4)	58.4 (3.11)	61.8 (4.0)
HFnu^o^	FD	HF power in normalized units: HF/(total power – VLF) × 100	43.2 (8.4)	41.6 (3.11)	38.2 (4.0)
LF/HF	FD	Ratio of LF/HF	1.43 (0.7)	1.42 (0.2)	1.65 (0.3)
Total power	FD	The variance of NN intervals over the temporal segment	4224.1 (2859.9)	4517.9 (2026.3)	4035.4 (1730.7)
VLF	FD	Spectral density power in the VLF range	1401.9 (1363.6)	1493.7 (765.4)	1416.4 (670.2)
SD1	NL^p^	The SD of the Poincaré plot perpendicular to the line of identity	48.68 (17.1)	50.7 (12.8)	45.7 (11.4)
SD2	NL	The SD of the Poincaré plot along the line of identity	113.2 (47.1)	185 (51.8)	215.6 (63.8)
SD2/SD1	NL	Ratio of SD2 and SD1	2.39 (0.78)	3.7 (0.8)	4.8 (1.1)

^a^BBI: beat-to-beat intervals.

^b^TD: time domain.

^c^SDNN: SD of normal-to-normal intervals.

^d^NN: normal-to-normal.

^e^RMSSD: root mean square of successive differences.

^f^PNN50: proportion of normal-to-normal intervals that differ by >50 milliseconds.

^g^SDANN: SD of the averages of normal-to-normal intervals

^h^N/A: not applicable.

^i^GM: geometric method.

^j^HF: high frequency.

^k^FD: frequency domain.

^l^LF: low frequency.

^m^LFnu: low frequency in normalized units.

^n^VLF: very low frequency.

^o^HFnu: high frequency in normalized units.

^p^NL: nonlinear.

As HRV features have different applications but are nevertheless correlated among themselves to varying degrees [84,89], we examined previous studies to select which features to include in our modeling. We started by selecting the three time domain features and one geometric method feature recommended by the Task Force of the European Society of Cardiology and the North American Society of Pacing and Electrophysiology [85]: SD of normal-to-normal intervals (SDNN), root mean square of successive differences (RMSSD), SDANN, and triangular index. As RMSSD and SD1 are identical, as are SDNN and SD2, we only entered RMSSD and SDNN in the models [84]. LF power in normalized units and HF power in normalized units are identical measures that capture the same information as LF/HF; therefore, we only included LF/HF in the models to estimate the ratio between SNS and PNS activity [84,90]. HF is also strongly correlated with PNN50 and RMSSD; therefore, we did not include it in the models. Despite eliminating SD1, SD2, HF, and LF, we decided to keep the ratios as SD2/SD1 and LF/HF as they could capture additional information compared with the individual measures [84]. The correlations among the final set of features across long-term (24 hours) and short-term (5 minutes) windows are shown in Tables 5 and 6. Finally, as HRV measurements explain different phenomena depending on the time window, we decided to use variance inflation factor (VIF) feature elimination [91] to determine the set of features for each particular model and time window and address concerns with multicollinearity.

**Table 5:** Repeated-measures correlations among the final set of features calculated during the 24 hours of the day when participants answered the surveys and 95% CI (N=14,695 observations from 657 participants).

Measures	Correlations, *r*_rm_ (95% CI)								
	SDNN^a^	RMSSD^b^	MRRI^c^	PNN50^d^	SDANN^e^	Triangular index	LF^f^/HF^g^	Total power	VLF^h^
SDNN	—^i^	—	—	—	—	—	—	—	—
RMSSD	0.2 (0.19 to 0.22)	—	—	—	—	—	—	—	—
MRRI	0.27 (0.25 to 0.28)	0.49 (0.48 to 0.51)	—	—	—	—	—	—	—
PNN50	0.21 (0.19 to 0.23)	0.93 (0.93 to 0.93)	0.45 (0.43 to 0.46)	—	—	—	—	—	—
SDANN	0.82 (0.81 to 0.82)	0.02 (0 to 0.03)	0.02 (0 to 0.04)	0.04 (0.03 to 0.06)	—	—	—	—	—
Triangular index	0.67 (0.66 to 0.68)	0.26 (0.24 to 0.27)	0.29 (0.28 to 0.31)	0.3 (0.29 to 0.32)	0.5 (0.49 to 0.51)	—	—	—	—
LF/HF	0.24 (0.23 to 0.26)	−0.33 (−0.34 to −0.31)	0.33 (0.31 to 0.34)	−0.35 (−0.37 to −0.34)	0.17 (0.15 to 0.18)	0.14 (0.12 to 0.16)	—	—	—
Total power	0.36 (0.35 to 0.38)	0.84 (0.83 to 0.84)	0.62 (0.61 to 0.63)	0.84 (0.84 to 0.84)	0.13 (0.11 to 0.14)	0.37 (0.36 to 0.38)	0.01 (−0.01 to 0.03)	—	—
VLF	0.42 (0.41 to 0.44)	0.69 (0.68 to 0.7)	0.63 (0.62 to 0.64)	0.68 (0.67 to 0.69)	0.18 (0.17 to 0.2)	0.39 (0.37 to 0.4)	0.15 (0.14 to 0.17)	0.94 (0.94 to 0.95)	—
SD2/SD1	0.69 (0.68 to 0.7)	−0.5 (−0.52 to −0.49)	−0.14 (−0.16 to −0.13)	−0.48 (−0.5 to −0.47)	0.67 (0.66 to 0.68)	0.37 (0.35 to 0.38)	0.47 (0.46 to 0.48)	−0.29 (−0.3 to −0.27)	−0.14 (−0.16 to −0.13)

^a^SDNN: SD of normal-to-normal intervals.

^b^RMSSD: root mean square of successive differences.

^c^MRRI: mean RR interval.

^d^PNN50: proportion of normal-to-normal intervals that differ by >50 milliseconds.

^e^SDANN: SD of the averages of normal-to-normal intervals.

^f^LF: low frequency.

^g^HF: high frequency.

^h^VLF: very low frequency.

^i^Upper triangle of the correlation matrix was omitted for simplicity and readability.

**Table 6:** Repeated-measures correlations among the final set of features calculated on the 5 minutes centered on the time when participants started answering the surveys and 95% CI (N=14,695 observations from 657 participants)^a^.

Measure	Correlations, *r*_rm_ (95% CI)						
	SDNN^b^	RMSSD^c^	MRRI^d^	PNN50^e^	LF^f^/HF^g^	Total power	VLF^h^
SDNN	—^i^	—	—	—	—	—	—
RMSSD	0.63 (0.62 to 0.64)	—	—	—	—	—	—
MRRI	0.27 (0.25 to 0.28)	0.61 (0.6 to 0.62)	—	—	—	—	—
PNN50	0.59 (0.58 to 0.6)	0.95 (0.94 to 0.95)	0.53 (0.52 to 0.54)	—	—	—	—
LF/HF	0.02 (0 to 0.03)	−0.11 (−0.13 to −0.1)	0.22 (0.2 to 0.24)	−0.15 (−0.16 to −0.13)	—	—	—
Total power	0.75 (0.74 to 0.76)	0.81 (0.8 to 0.81)	0.55 (0.54 to 0.57)	0.73 (0.72 to 0.74)	0.13 (0.11 to 0.14)	—	—
VLF	0.73 (0.72 to 0.74)	0.53 (0.52 to 0.54)	0.34 (0.32 to 0.35)	0.46 (0.45 to 0.47)	0.12 (0.1 to 0.13)	0.88 (0.88 to 0.88)	—
SD2/SD1	0.52 (0.51 to 0.53)	−0.27 (−0.29 to −0.26)	−0.28 (−0.3 to −0.27)	−0.28 (−0.29 to −0.26)	0.24 (0.22 to 0.25)	0.05 (0.03 to 0.07)	0.28 (0.27 to 0.3)

^a^SD of the averages of normal-to-normal intervals and triangular index are not included as they should not be calculated in a single 5-minute time window.

^b^SDNN: SD of normal-to-normal intervals.

^c^RMSSD: root mean square of successive differences.

^d^MRRI: mean RR interval.

^e^PNN50: proportion of normal-to-normal intervals that differ by >50 milliseconds.

^f^LF: low frequency.

^g^HF: high frequency.

^h^VLF: very low frequency.

^i^Upper triangle of the correlation matrix was omitted for simplicity and readability.

### Data Exclusion

To account for missing EMA or smartwatch data during both studies (eg, dead battery or device not worn), days were excluded from the sample if any value was missing from the predictors for that day. This resulted in a final data set of 14,695 entries in the main study and 1373 in the follow-up study of matching psychological and physiological measures.

### HRV Analysis

#### Main Study

The main purpose of this study was to examine the relationship between HRV and perceived stress as assessed by a daily stress survey. Many of the HRV features calculated are suited for short time frame measurements (eg, 2 minutes), as well as the long term (eg, 24 hours); however, Shaffer and Ginsberg [84] cautioned that these are not to be used interchangeably. Therefore, given the conflicting evidence presented in the related works as to when it is best to measure HRV in relation to a stressful event, we tested a series of models for predicting the daily stress survey response, with HRV features derived (1) 5 minutes before completing the survey, (2) 30 minutes before, (3) 5 minutes after, (4) 30 minutes after, (5) using time windows of varying length (5 minutes, 30 minutes, 1 hour, 2 hours, 4 hours, 8 hours, and 24 hours) centered on the moment the survey was started, (6) during the entire 24 hours on the day a participant answered the survey, and (7) during the “work day” from 8 AM to 6 PM. For sake of brevity, we report all the coefficients only for the model using the time frame with the best fit in the main results, whereas the coefficients of the models across all other time windows are reported in the form of density plots in Figure S5 in Multimedia Appendix 2. Finally, we examined the overall variance explained in the outcome measure of daily perceived stress from the HRV features.

To determine whether HRV specifically predicts stress or simply indicates arousal, which correlates with other psychological measures, we first built models to examine whether our derived HRV features predicted other survey measures that are known to have a relationship with psychological stress or arousal: positive affect, negative affect, and anxiety [92,93]. Then, to understand whether there is specificity in predicting perceived stress, we further built two models: a model predicting stress using anxiety, positive affect, and negative affect as predictors, and a second model incorporating HRV as an additional predictor.

#### Follow-up Study

In the analysis conducted in the follow-up study, we leveraged the additional information gained from participants related to their perceived stress duration and evaluated how well the HRV features can predict perceived stress at the reported most stressful time of the day and again predict perceived stress at the time of survey response (the same question asked in the main study). For predicting perceived stress at the reported most stressful time of the day and perceived stress at the time of survey response, in this study, we computed the HRV features in the same manner as in the main study and used the best performing time window found earlier while also considering HRV features calculated during participants’ reported most stressful periods for that day. We proceeded to compare these 2 models in predicting both perceived stress at the reported most stressful time of the day and perceived stress at the time of survey response. In addition, we considered the duration of perceived stress at the reported most stressful time of the day as an outcome measure in itself to better understand whether HRV is related to the saliency (score) of the stress events or the duration.

### Modeling Strategy

As our data comprises repeated observations for each participant, and stress and anxiety are ordinal variables, we used cumulative link mixed-effects models [94] using a random intercept for the participant. We considered using random slopes in our models but decided against it because of model convergence issues in the main study and not having enough observations to support such random effects structure in the follow-up study. In the cases of predicting positive affect and the duration of perceived stress at the reported most stressful time of the day (follow-up study), we used linear mixed-effects models [95,96] as the variables can be considered continuous. In the case of negative affect, we used a negative binomial generalized linear mixed-effects model, given the distribution of the variable (Figure 1). As stated earlier, we used VIF [97] feature elimination to iteratively remove VIFs >3 to address multicollinearity [91,98]. As the predictors were on vastly different scales, all predictor variables were *z* score standardized before being entered into the models. Pseudo *R*^2^ values for both marginal (fixed effects alone) and conditional (random and fixed) effects are reported using the method described by Nakagawa and Schielzeth [99].

### Ethics Approval

The study protocol was approved by the University of Notre Dame Institutional Review Board (17-5-3870).

## Results

### Main Study

**Figure 4:**
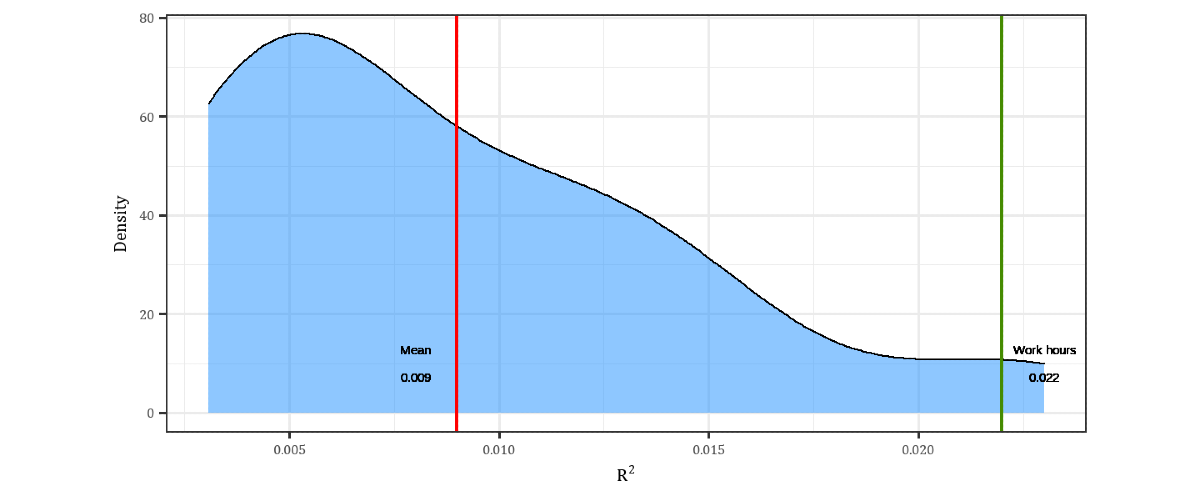
Density plot of marginal R2 across time windows from 5 minutes to 24 hours.

Figure 4 provides a density plot of the variance explained (pseudo *R*^2^) by the HRV features across all periods. On average, HRV explained a small portion (approximately 1%) of the variability in perceived stress. We also found that the model with features computed during the hours of 8 AM to 6 PM had the lowest Akaike information criterion (AIC) and explained the highest variance (Figure 4), although this was still modest (2.2%). Coefficients for this model are reported in Table 7, whereas density plots of coefficients across all time windows are included in Figure S5 (Multimedia Appendix 2).

Regarding whether HRV predicts perceived stress specifically or simply predicts arousal, we found that the directionality of most of the associations was the same for stress, anxiety, positive affect, and negative affect (Tables 7 and 8). Mean RR interval was a significant predictor of anxiety and positive affect but was not significant in predicting stress. LF to HF ratio and triangular index were both significant predictors of stress; however, LF to HF ratio was not a significant predictor of negative affect, and triangular index was not a significant predictor of positive affect.

In addition, after controlling for positive affect, negative affect, and anxiety, most HRV features were still significant predictors of perceived stress, and when compared against a model that only considers the measures of affect and anxiety, a model containing HRV provided a better fit (Table 7), as confirmed by likelihood ratio tests and AIC (*χ*^2^_5_=157.8; *P*<.001; AIC 23,561 vs 23,709).

**Table 7:** Model for perceived stress with variance inflation factor–reduced HRV^a^ features derived from beat-to-beat interval data during normal work hours of 8 AM to 6 PM^b^.

Predictors	Perceived stress at the time of survey response from HRV^c^			Perceived stress at the time of survey response from anxiety, positive affect, and negative affect^d^			Perceived stress at the time of survey response from anxiety, positive affect, negative affect, and HRV^e^	
	OR^f^ (95% CI)	*P* value	OR (95% CI)		*P* value	OR (95% CI)		*P* value
MRRI^g^	0.95 (0.89-1.02)	.16	—^h^		—	1.01 (0.94-1.09)		.75
LF^i^/HF^j^	0.86 (0.82-0.91)	<.*001*^k^	—		—	0.85 (0.81-0.90)		*<.001* ^k^
VLF^l^	1.54 (1.42-1.67)	*<.001* ^k^	—		—	1.31 (1.20-1.43)		*<.001* ^k^
Triangular index	0.88 (0.83-0.94)	*<.001* ^k^	—		—	0.94 (0.88-1.01)		.10
SDANN^m^	0.74 (0.69-0.78)	*<.001* ^k^	—		—	0.81 (0.76-0.86)		*<.001* ^k^
Anxiety	—	—	5.38 (5.05-5.73)		*<.001* ^k^	5.30 (4.97-5.64)		*<.001* ^k^
Positive affect	—	—	0.96 (0.91-1.01)		.11	0.94 (0.89-0.99)		*.01*
Negative affect	—	—	2.52 (2.37-2.68)		*<.001* ^k^	2.53 (2.38-2.69)		*<.001* ^k^

^a^HRV: heart rate variability.

^b^Model fit on 14,695 observations from 657 participants. Cumulative link mixed-effects model thresholds are omitted for brevity. An extended version with threshold values is available in Table S15 in Multimedia Appendix 5.

^c^Random effects: σ^2^=3.29; τ_00_=2.25; participant intraclass correlation coefficient 0.41; marginal *R*^2^/conditional *R*^2^=0.022/0.420; Akaike information criterion 31,602.

^d^Random effects: σ^2^=3.29; τ_00_=1.48; participant intraclass correlation coefficient 0.31; marginal *R*^2^/conditional *R*^2^=0.547/0.688; Akaike information criterion 23,709.

^e^Random effects: σ^2^=3.29; τ_00_=1.52; participant intraclass correlation coefficient 0.32; marginal *R*^2^/conditional *R*^2^=0.548/0.691; Akaike information criterion 23,561.

^f^OR: odds ratio.

^g^MRRI: mean RR interval.

^h^The predictor was not included in this model.

^i^LF: low frequency.

^j^HF: high frequency.

^k^*P* values lower than .05 are highlighted in italics.

^l^VLF: very low frequency.

^m^SDANN: SD of the averages of normal-to-normal intervals.

**Table 8:** Model for anxiety (cumulative link mixed-effects model) and negative affect (linear mixed-effects model) with variance inflation factor–reduced heart rate variability features derived from beat-to-beat interval data during normal work hours of 8 AM to 6 PM^a^.

Predictors	Positive affect^b^				Negative affect^c^				Anxiety^d^	
	Standardized β	Standardized 95% CI	*P* value	IRR^e^		95% CI	*P* value	OR^f^ (95% CI)		*P* value
Intercept	−.01	−0.07 to 0.05	*<.001*	6.32		6.22 to 6.43	*<.001*	—^g^		—
MRRI^h^	−.15	−0.17 to −0.12	*<.001*	0.99		−0.06 to −0.004	.06	0.90 (0.83 to 0.97)		*.004* ^i^
LF^j^/HF^k^	−.08	−0.10 to −0.07	*<.001*	1.00		−0.03 to 0.01	.88	0.92 (0.87 to 0.97)		*.002* ^i^
VLF^l^	.12	0.09 to 0.15	*<.001*	1.04		0.06 to 0.12	*<.001*	1.51 (1.39 to 1.65)		*<.001* ^i^
Triangular index	.00	−0.02 to 0.02	.91	0.98		−0.08 to −0.03	*.001*	0.91 (0.85 to 0.97)		*.005* ^i^
SDANN^m^	−.03	−0.05 to −0.01	*.002*	0.98		−0.07 to **−**0.03	*<.001*	0.76 (0.72 to 0.81)		*<.001* ^i^

^a^Models fit on 14,695 observations from 657 participants. *P* values <.05 are highlighted in italics. Cumulative link mixed-effects model thresholds are omitted for brevity. An extended version with threshold values is available in Table S16 in Multimedia Appendix 5.

^b^Random effects: σ^2^=9.03; τ_00_=9.69; participant intraclass correlation coefficient 0.52; marginal *R*^2^/conditional *R*^2^=0.020/0.527.

^c^Random effects: σ^2^=0.15; τ_00_=0.03; participant intraclass correlation coefficient 0.19; marginal *R*^2^/conditional *R*^2^=0.004/0.191.

^d^Random effects: σ^2^=3.29; τ_00_=2.51; participant intraclass correlation coefficient 0.43; marginal *R*^2^/conditional *R*^2^=0.015/0.441.

^e^IRR: incidence rate ratio.

^f^OR: odds ratio.

^g^The predictor was not included in this model.

^h^MRRI: mean RR interval.

^i^*P* values lower than .05 are highlighted in italics.

^j^LF: low frequency.

^k^HF: high frequency.

^l^VLF: very low frequency.

^m^SDANN: SD of the averages of normal-to-normal intervals.

### Follow-up Study

We first assessed whether using the context provided by participants to determine an HRV window to calculate the features provided a benefit over the previously found best time window of work hours of the day. Our outcome variables were perceived stress at the time of survey response and perceived stress at the reported most stressful time of the day. In the case of perceived stress at the time of survey response, the model of HRV during work hours (reported in Table 9) achieved the best fit with an *R*^2^ of 0.032 versus 0.022 and AIC of 3465 versus 3475, therefore favoring the model with HRV features calculated during the workday, as in the main study. It also replicates findings from the main study, which found an *R*^2^ of 0.022. Similar results are obtained when predicting perceived stress at the reported most stressful time of the day (*R*^2^ of 0.023 vs 0.015), with the model based on HRV during work hours reported in Table 9 and a full comparison available in Tables S11 to S12 in Multimedia Appendix 3. Thus, we did not observe benefits from computing HRV features based on self-reported most stressful time of the day compared with the entire workday. We also found that HRV during work hours was predictive of the duration of perceived stress at the reported most stressful time of the day (Table 9), although the fit was quite small.

As the duration of perceived stress at the reported most stressful time of the day was correlated with perceived stress at the time of survey response and perceived stress at the reported most stressful time of the day scores (Table 3), we conducted a post hoc analysis to investigate whether HRV could predict the saliency of the perceived stress while controlling for the effects of the duration of the event and elapsed time since it occurred—contextual features provided through self-report. Including HRV features along with contextual features provided a better fit (*R*^2^ of 0.064 vs 0.050) over simply using the contextual features. This was further confirmed by likelihood ratio tests and AIC (*χ*^2^_5_=22.9; *P*<.001; AIC 3242 vs 3255; see Tables S13 and S14 in Multimedia Appendix 4 for the full models).

**Table 9:** Prediction of perceived stress at the time of survey response, perceived stress at the reported most stressful time of the day, and duration of perceived stress at the reported most stressful time of the day with the same predictors—heart rate variability during work hours—as in the best model in the main study^a^.

Predictors	Perceived stress at the time of survey response^b^			Perceived stress at the reported most stressful time of the day^c^			Duration of perceived stress at the reported most stressful time of the day^d^		
	OR^e^	*P* value	OR		*P* value	β		95% CI	*P* value
Intercept	—^f^	—	—		—	.02		−0.06 to 0.10	.64
MRRI^g^	0.98 (0.79 to 1.23)	.89	0.86 (0.70 to 1.07)		.18	−.03		−0.12 to 0.07	.59
LF^h^/HF^i^	0.84 (0.73 to 0.98)	*.03* ^j^	0.85 (0.73 to 0.99)		*.04*	−.02		−0.09 to 0.05	.57
VLF^k^	1.56 (1.22 to 1.99)	*<.001* ^j^	1.54 (1.21 to 1.97)		*<.001*	.15		0.05 to 0.25	*.005*
Triangular index	0.79 (0.63 to 0.99)	*.04* ^j^	0.98 (0.79 to 1.24)		.93	−.11		−0.19 to −0.03	*.03*
SDANN^l^	0.75 (0.61 to 0.91)	*.003* ^j^	0.73 (0.60 to 0.89)		*.002*	−.10		−0.20 to −0.01	*.008*

^a^The models were fit with 1373 observations from 327 participants. Cumulative link mixed-effects models threshold values are omitted for brevity. An extended version with threshold values is available in Table S17 in Multimedia Appendix 5.

^b^Random effects: σ^2^=3.29; τ_00_=1.21; participant intraclass correlation coefficient 0.27; marginal *R*^2^/conditional *R*^2^=0.032/0.292.

^c^Random effects: σ^2^=3.29; τ_00_=0.97; participant intraclass correlation coefficient 0.23; marginal *R*^2^/conditional *R*^2^=0.023/0.245.

^d^Random effects: σ^2^=0.60: τ_00_=0.40; participant intraclass correlation coefficient 0.40; marginal *R*^2^/conditional *R*^2^=0.019/0.414.

^e^OR: odds ratio.

^f^Cumulative Link Mixed Models have multiple thresholds rather than one intercept. Therefore, no value for an intercept is included in this table.

^g^MRRI: mean RR interval.

^h^LF: low frequency.

^i^HF: high frequency.

^j^*P* values lower than .05 are highlighted in italics.

^k^VLF: very low frequency.

^l^SDANN: SD of the averages of normal-to-normal intervals.

## Discussion

### Principal Findings

Stress is associated with many negative outcomes [3-6], thereby making accurate measurement and management of it an important aspect of improving both physical and mental health outcomes. To this end, the ubiquitous computing and mobile health communities have turned to wearables and, more specifically, identified wearable-sensed HRV as an attractive method for passively sensing stress [12,23,24,29]. However, does the evidence support associating HRV—as measured with wearables in the wild—with stress, as perceived by the user?

We found that the best model yielded a marginal *R*^2^ of 2.2%, which approximately corresponds to a correlation of 0.15 and a Cohen *d* of 0.30, which lies between a small (Cohen *d*=0.20) to medium (Cohen *d*=0.50) effect [100,101]. Thus, HRV was weakly, although significantly, associated with perceived stress when measured using a wearable in naturalistic settings. The size of this effect is, to some degree, expected, given that HRV only measures ANS activity and not HPA activity, thus being an incomplete assessment of stress, even in ideal conditions. That said, we would have expected a stronger relationship between perceived stress and HRV a priori, given its popular use in assessing stress [8,34-37]. Nevertheless, despite the small magnitude of the effect, we also found some evidence for incremental prediction in that HRV uniquely predicted perceived stress above and beyond self-reported positive affect, negative affect, and anxiety (Table 7).

We do not believe the small effect size is because of how perceived stress was assessed, as using validated assessments of related constructs, such as negative affect and anxiety, yielded similar results (Table 8) and was highly correlated with stress (Table 2). Our findings suggest that the signal provided by wearable-measured HRV is of limited use in predicting perceived stress in the wild in the absence of clear and isolated stressors (such as those provided in laboratory studies).

Regarding the optimal temporal association between HRV and perceived stress, we found that HRV features measured around the time of the survey response—when participants were assessing their current stress level—yielded a lower fit than a generic time window covering the workday (ie, between 8 AM to 6 PM). This is different from the results in laboratory settings, which suggest the optimal time window to be shorter and closer to the assessment of stress, given the quick SNS response to induced stress. Although the length of the time window in which HRV is measured can affect what contributes to the changes in the HRV features (eg, circadian rhythms might be captured with longer-term HRV but not short term [84]), the estimates found within the “workday” time window of 8 AM to 6 PM were generally consistent in directionality with previous literature for changes in HRV because of stress.

Specifically, triangular index and SDANN were both negatively associated with perceived stress. Both of these match the expectation that lower HRV would indicate higher stress [29]. VLF was positively associated with perceived stress, which is to be expected as SNS activity because of stress (among other reasons) modulates the amplitude and frequency of HRV measured in this band [84,102]. Finally, the ratio of LF to HF was negatively associated with perceived stress in the work hours time window, which might be considered counterintuitive. In controlled conditions, LF/HF can be used as a measure of autonomic balance; that is, it is assumed that PNS and SNS activity contributes to LF, and PNS largely contributes to HF [84]. Therefore, one could have expected a higher LF/HF ratio to equate to higher perceived stress, as it would indicate more SNS than PNS activity. Nevertheless, as highlighted in the study by Shaffer and Ginsberg [84], because of the complex relationship between SNS and PNS activity, LF/HF ratio will not always index autonomic balance. Thus, it is possible that in the conditions of this study, either a higher LF/HF was an indicator of higher PNS activity over SNS activity, or a higher PNS activity was a better marker for the saliency of a previous stressful event from which the participant was recovering at the time of the survey response.

In the follow-up study, our modified stress survey aimed to identify and compute HRV based on participants’ most stressful time of the day. Although this is impractical for a real-world use case, it does allow measurement of HRV closer to the stressor, as in many laboratory studies. Nevertheless, measuring HRV during the most stressful time of the day yielded a lower model fit than using the generic 8 AM to 6 PM time window (Multimedia Appendix 3). Therefore, we believe the small effect of HRV as a predictor of stress ostensibly resides in the conditions of measurement themselves. Specifically, in laboratory-based studies, the measurements of changes in HRV because of stress occur in the presence of clear and isolated stressors (eg, stress being induced by the study conditions, causing an increase in SNS activity), which, in turn, implies that HRV changes because stress, and these changes can often cease with the end of a stressor [51]. Discrete and isolated stressors in controlled laboratory studies may not be as common in naturalistic settings, making results from these studies under controlled conditions not fully applicable to daily life settings.

In naturalistic settings, identifying perceived stress at the precise moment of a clear and isolated stressor would be difficult to achieve from HRV alone for several reasons. First, physiological stress is different from perceived stress. For instance, physical exertion or exercise is generally classified as a physiological stressor (and would exhibit increases in HR, decreased HRV, and increases in cortisol); however, it is well known that exercise can reduce perceived stress [103] and generally would not be reported as *stressful* by participants. Second, self-reports are subject to emotional perception and expression biases [104-107], as well as memory biases and/or coping responses [73,74]. Finally, EMAs are designed to measure stress at either random or specific times, although participants may not respond at the designated time (eg, at the end of a stressor as opposed to the middle of a stressor).

In summary, our main conclusion is that the reported association between HRV and perceived stress may depend on laboratory conditions. In naturalistic studies, there are no clear and direct links between isolated stressors and SNS responses. Although there is still an observable association between wearables and perceived stress, it is weak, and it suggests that HRV alone should not be considered a valid proxy measure of perceived stress in naturalistic studies.

### Implications of This Study

Although HRV has been shown to be a useful biomarker of perceived stress in laboratory studies, we have shown that in the wild, perceived stress does not always align strongly with physiological stress. This is of special importance as an increasing number of studies and commercial applications in the ubiquitous computing community use wearables to measure stress using HRV, sometimes under the assumption that there is a very strong alignment between the two, when, in fact, the alignment is more tenuous. Although it is beneficial to have wearables capable of providing continuous measurement of HRV unobtrusively, we caution against the use of HRV features as sole or main indicators of “stress” in user-facing applications, as the results may not align with perceived stress. This level of inaccuracy risks an increase of distrust in health and well-being applications at a minimum. It can have more profound negative effects as well, and based on the present findings, labeling HRV as “stress” without proper validity data would be highly suspect. Therefore, we would encourage future work in the scientific community to investigate complementary sensing streams that could serve as markers of stress and use those in conjunction with HRV.

To realize the goal of monitoring the health of individuals, such sensing streams should be rigorously vetted through longitudinal studies to appropriately measure their predictive power for capturing intraindividual differences over time. Nevertheless, it is unlikely that any single physiological sensing stream would be able to perfectly align with perceived stress. Therefore, rather than looking at a single biomarker of the ANS, as is HRV, a more complete view of the ANS response could perhaps delineate a viable strategy for health monitoring unobtrusively in the wild. More broadly, approaches based on multimodality are more likely to yield successful outcomes in health monitoring, as recent studies show in other fields such as sleep monitoring [108], job performance monitoring [109,110], and personality prediction [111].

### Limitations

It is important to note that this study has limitations. First, our sample comprised information workers who might be less likely to have movement artifacts that could affect the wearable measurements of HRV. Second, our sample was fairly homogenous, with participants whose income and education levels were above the US average (low-income and lower education populations were underrepresented). Third, we are unable to determine the accuracy of self-reported stress durations and timing of stress. Similarly, the duration of the most stressful time of the day was correlated with the perceived stress at that time, and it is possible that participants’ response to one question influenced the answer to the other (ie, judging stressors that last longer as more intense). Finally, the items introduced in the follow-up study were not validated in this or other studies. Addressing these limitations is a goal for future work.

### Conclusions

We examined the alignment of physiological stress (HRV), as measured with a consumer-grade wearable device, and perceived stress in an 8-week study with information workers from multiple organizations across the United States. We found a weak but significant association between HRV and perceived stress, which was replicated in a week-long follow-up study a year later. Computing HRV across the workday outperformed other time windows, including self-reported stressful events. Overall, our findings suggest that wearable-based HRV should not be used as a sole biomarker for perceived stress in naturalistic settings. Instead, it might best be used in conjunction with other measures to measure this complex phenomenon in the wild.

## Appendix

Multimedia Appendix 1 Correlation of the stress item with other validated measures.

Multimedia Appendix 2 Summary of odds ratios of predictors across different time windows.

Multimedia Appendix 3 Comparison between models, including heart rate variability calculated during the workday and during the reported most stressful time of the day.

Multimedia Appendix 4 Additive value of heart rate variability features over considering only the participants’ subjective assessment.

Multimedia Appendix 5 Expanded versions of the tables included in the main paper, including the threshold values for the ordinal models.

## Abbreviations

AIC: Akaike information criterion
ANS: autonomic nervous system
BBI: beat-to-beat intervals
ECG: electrocardiogram
EMA: ecological momentary assessment
HF: high frequency
HPA: hypothalamic-pituitary-adrenal
HR: heart rate
HRV: heart rate variability
LF: low frequency
PNS: parasympathetic nervous system
RMSSD: root mean square of successive differences
SDANN: SD of the averages of normal-to-normal intervals
SDNN: SD of normal-to-normal intervals
SNS: sympathetic nervous system
VIF: variance inflation factor
VLF: very low frequency
