# Supplementary material for: Alignment Between Heart Rate Variability From Fitness Trackers and Perceived Stress: Perspectives From a Large-Scale In Situ Longitudinal Study of Information Workers
Source: JMIR Hum Factors. 2022 Aug 4;9(3):e33754. doi: 10.2196/33754 (PMC9389384; doi:10.2196/33754)
Supplement: Multimedia Appendix 1 [file humanfactors_v9i3e33754_app1.docx]

## Multimedia Appendix 1: Correlation of the stress item with other validated measures

Table S10: Correlation of the Stress item with other measures from an unpublished study [76] of 991 Mechanical Turk Participants

| Measure | Pearson r |
| --- | --- |
|  |  |
| Mini-IPIP Neuroticism | .51 |
| IPIP-NEO-300 Neuroticism | .55 |
| Individual Task Proficiency | -.20 |
| Counterproductive Work Behavior – Checklist | .20 |
| Pittsburgh Sleep Quality Inventory | .42 |
| State Anxiety | .58 |
| Trait Anxiety | .61 |
| PANAS Positive Affect | -.33 |
| PANAS Negative Affect | .55 |
| Interpersonal Deviance | .12 |
| Organizational Deviance | .23 |
| IPIP-NEO-300 Anxiety | .58 |
| IPIP-NEO-300 Anger | .47 |
| IPIP-NEO-300 Depression | .55 |
