# Supplementary material for: Alignment Between Heart Rate Variability From Fitness Trackers and Perceived Stress: Perspectives From a Large-Scale In Situ Longitudinal Study of Information Workers
Source: JMIR Hum Factors. 2022 Aug 4;9(3):e33754. doi: 10.2196/33754 (PMC9389384; doi:10.2196/33754)

## Multimedia Appendix 2: Summary of ORs of predictors across different time windows

Figure S5: Odds ratios for predictors across models with HRV features created across different time windows, from 5 minutes to 24 hours.


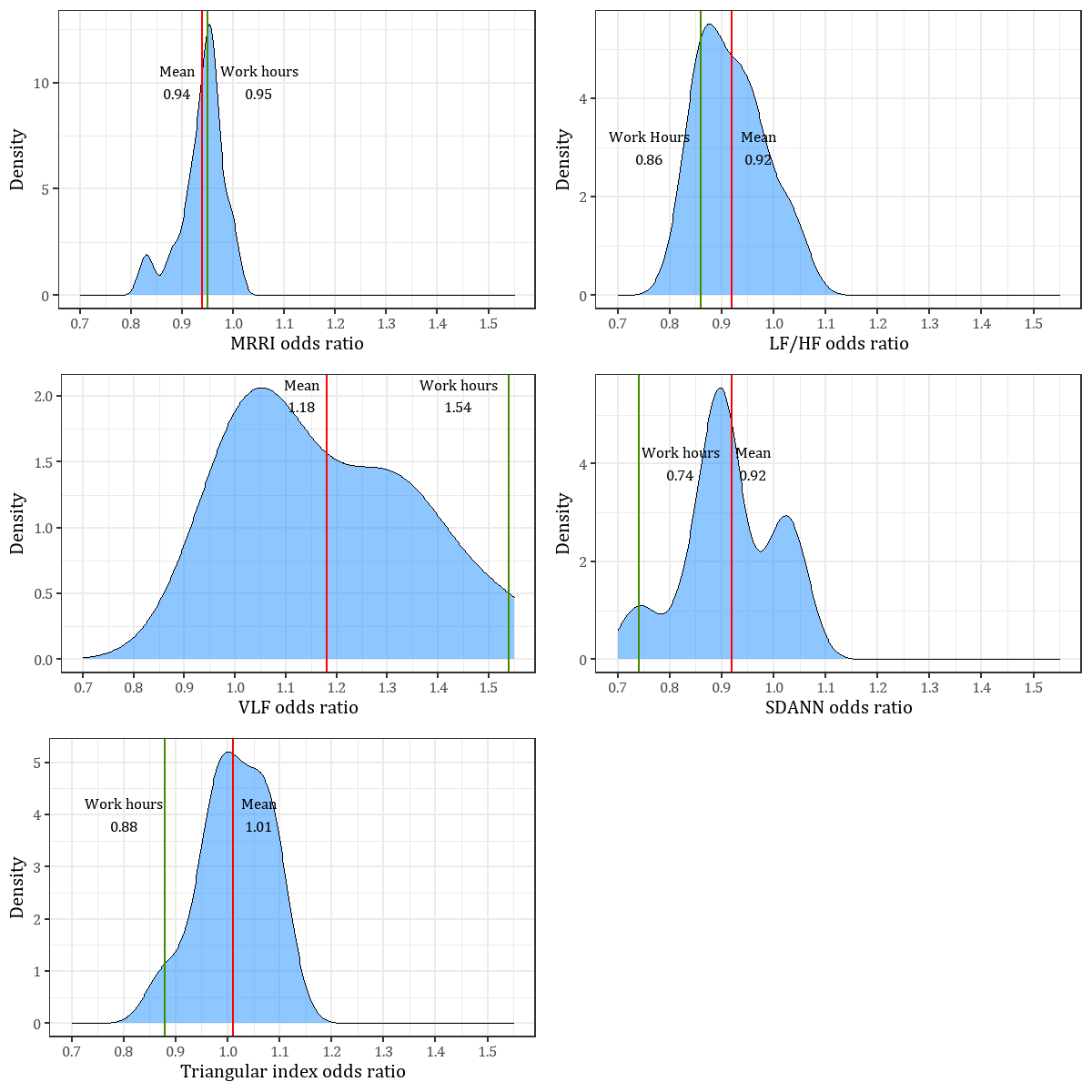

Supplement: Multimedia Appendix 2 [file humanfactors_v9i3e33754_app2.docx]
