# Supplementary material for: Alignment Between Heart Rate Variability From Fitness Trackers and Perceived Stress: Perspectives From a Large-Scale In Situ Longitudinal Study of Information Workers
Source: JMIR Hum Factors. 2022 Aug 4;9(3):e33754. doi: 10.2196/33754 (PMC9389384; doi:10.2196/33754)
Supplement: Multimedia Appendix 3 [file humanfactors_v9i3e33754_app3.docx]

**Multimedia Appendix 3: Comparison between models including HRV calculated during the work day and during the reported most stressful time of the day.**

Table S11: Comparison in the prediction of PSTR of a model with the same predictors as in the best model in the main study and one with HRV calculated during the reported most stressful time of the day. The models are fit on 1,373 observations from 327 participants.

| Predictors | Perceived stress at the time of survey response predicted from HRV calculated during the work day (8AM to 6PM)^a^ | | | Perceived stress at the time of survey response predicted from HRV calculated during the reported most stressful time of the day ^b^ | | |
| --- | --- | --- | --- | --- | --- | --- |
|  | Estimate / OR | CI | *P* | Estimate / OR | CI | *P* |
|  |  |  |  |  |  |  |
| 1\|2^c^ | -2.16 | -2.38 – -1.94 | *<.001* | -2.14 | -2.36 to -1.92 | *<.001* |
| 2\|3^c^ | 0.18 | 0.01 – 0.35 | *.041* | 0.17 | 0.00 – 0.34 | .052 |
| 3\|4^c^ | 2.52 | 2.28 – 2.75 | *<.001* | 2.48 | 2.25 – 2.72 | *<.001* |
| 4\|5^c^ | 2.71 | 2.46 – 2.96 | *<.001* | 2.68 | 2.43 – 2.92 | *<.001* |
| MRRI | 0.98 | 0.79 – 1.23 | .889 | 0.98 | 0.81 – 1.20 | .880 |
| LF/HF | 0.84 | 0.73 – 0.98 | *.029* | 0.98 | 0.86 – 1.12 | .773 |
| VLF | 1.56 | 1.22 – 1.99 | *<.001* | 0.83 | 0.69 – 1.00 | *.048* |
| Tri-index | 0.79 | 0.63 – 0.99 | *.039* | 1.52 | 1.28 – 1.81 | *<.001* |
| SDANN | 0.75 | 0.61 – 0.91 | *.003* | **-** | **-** | **-** |
| SD2/SD1 | - | **-** | - | 0.89 | 0.76 – 1.03 | .116 |

^a^ Random Effects: σ^2^ = 3.29, τ_00_ =1.21 _participant_, ICC = 0.27,
Marginal R^2^ / Conditional R^2^ = 0.032 / 0.292. AIC = 3465.

^b^ Random Effects: σ^2^ = 3.29, τ_00_ =1.13 _participant_, ICC = 0.25,
Marginal R^2^ / Conditional R^2^ = 0.022 / 0.272. AIC = 3476.

^c^Estimates are reported for Threshold values instead of odds ratios.

Table S12: Comparison in the prediction of PSMS of a model with the same predictors as in the best model in the main study and one with HRV calculated during the reported most stressful time of the day. The models are fit on 1,373 observations from 327 participants.

| Predictors | Perceived stress at the reported most stressful time of the day predicted from HRV calculated during the work day (8AM to 6PM)^a^ | | | Perceived stress at the reported most stressful time of the day from HRV calculated during the reported most stressful time of the day ^b^ | | |
| --- | --- | --- | --- | --- | --- | --- |
|  | Estimate /OR | CI | *P* | Estimate /OR | CI | *P* |
|  |  |  |  |  |  |  |
| 1\|2^c^ | -4.14 | -4.52 to -3.75 | *<.001* | -4.12 | -4.51 to -3.74 | *<.001* |
| 2\|3^c^ | -1.60 | -1.79 to -1.40 | *<.001* | -1.59 | -1.78 to -1.40 | *<.001* |
| 3\|4^c^ | 1.28 | 1.10 – 1.46 | *<.001* | 1.27 | 1.09 – 1.45 | *<.001* |
| 4\|5^c^ | 1.62 | 1.43 – 1.81 | *<.001* | 1.61 | 1.42 – 1.80 | *<.001* |
| MRRI | 0.86 | 0.70 – 1.07 | .184 | 0.82 | 0.67 – 1.00 | *.04****7*** |
| LF/HF | 0.85 | 0.73 – 0.99 | *.036* | 1.00 | 0.87 – 1.15 | .995 |
| VLF | 1.54 | 1.21 – 1.97 | *<.001* | 0.95 | 0.81 – 1.11 | .500 |
| Tri-index | 0.99 | 0.79 – 1.24 | .932 | 1.38 | 1.16 – 1.63 | *<.001* |
| SDANN | 0.73 | 0.60 – 0.89 | *.002* | **-** | - | **-** |
| SD2/SD1 | - | **-** | - | 0.80 | 0.69 – 0.92 | *.002* |

^a^ Random Effects: σ^2^ = 3.29, τ_00_ =0.97 _participant_, ICC = 0.23,
Marginal R^2^ / Conditional R^2^ = 0.023 / 0.245. AIC = 3268.

^b^ Random Effects: σ^2^ = 3.29, τ_00_ =0.94 _participant_, ICC = 0.22,
Marginal R^2^ / Conditional R^2^ = 0.015 / 0.233. AIC = 3275.

^c^Estimates are reported for Threshold values instead of odds ratios.
