# Supplementary material for: Alignment Between Heart Rate Variability From Fitness Trackers and Perceived Stress: Perspectives From a Large-Scale In Situ Longitudinal Study of Information Workers
Source: JMIR Hum Factors. 2022 Aug 4;9(3):e33754. doi: 10.2196/33754 (PMC9389384; doi:10.2196/33754)
Supplement: Multimedia Appendix 4 [file humanfactors_v9i3e33754_app4.docx]

**Multimedia Appendix 4: Additive value of HRV features over considering only the participants’ subjective assessment.**

Table S13. Models predicting perceived stress at the time of survey response (PSTR) using the contextual features and HRV. 1,373 observations of 327 participants. Lower AIC and a likelihood ratio test (P < .001) show that HRV features do provide additive value over only considering context variables. HRV predicted the saliency of the PSTR while controlling for self-reported duration of the most stressful time of the day (duration of PSMS) and the length of time between when the participants answered the survey and when their self-reported most stressful time of the day happened.

| Predictors | PSTR^a^ | | | PSTR^b^ | | |
| --- | --- | --- | --- | --- | --- | --- |
|  | Estimate / OR | OR CI | *P* | Estimate / OR | OR CI | *P* |
|  |  |  |  |  |  |  |
| 1\|2^c^ | -2.34 | -2.57 to -2.12 | *<.001* | -2.37 | -2.59 to -2.14 | *<.001* |
| 2\|3^c^ | 0.13 | -0.04 – 0.31 | .216 | 0.15 | -0.02 – 0.32 | .177 |
| 3\|4^c^ | 2.77 | 2.52 – 3.02 | *<.001* | 2.81 | 2.55 – 3.06 | *<.001* |
| 4\|5^c^ | 2.99 | 2.72 – 3.25 | *<.001* | 3.03 | 2.76 – 3.30 | *<.001* |
| Duration of PSMS | 2.10 | 1.80 – 2.44 | *<.001* | 1.99 | 1.71 – 2.32 | *<.001* |
| Time between the start of the survey and the end of the most stressful time | 0.64 | 0.56 – 0.74 | *<.001* | 0.62 | 0.54 – 0.72 | *<.001* |
| MRRI | - | - | - | 0.98 | 0.79 – 1.22 | .886 |
| LF/HF | - | - | - | 0.84 | 0.72 – 0.98 | *.026* |
| VLF | - | - | - | 1.47 | 1.15 – 1.89 | *.002* |
| Tri-index | - | - | - | 0.86 | 0.68 – 1.07 | .180 |
| SDANN | - | - | - | 0.80 | 0.66 – 0.97 | *.025* |

^a^ RE: σ^2^ = 3.29, τ_00_ = 1.03 _participant_, ICC = 0.24,
Marginal R^2^ / Conditional R^2^ = 0.199 / 0.390. AIC = 3255.

^b^ RE: σ^2^ = 3.29, τ_00_ = 1.06 _participant_, ICC = 0.24,
Marginal R^2^ / Conditional R^2^ = 0.213 / 0.404. AIC = 3242.

^c^Estimates are reported for Threshold values instead of odds ratios.

Table S14. Models predicting Perceived stress at the reported most stressful time of the day (PSMS) using the contextual features and HRV. 1,373 observations of 327 participants. Lower AIC and a likelihood ratio test (P = .002) show that HRV features do provide additive value over only considering context variables. HRV predicted the saliency of the PSMS while controlling for self-reported duration of the most stressful time of the day (duration of PSMS) and the length of time between when the participants answered the survey and when their self-reported most stressful time of the day happened.

| Predictors | PSMS^a^ | | | PSMS^b^ | | |
| --- | --- | --- | --- | --- | --- | --- |
|  | Estimate / OR | CI | *P* | Estimate / OR | CI | *P* |
|  |  |  |  |  |  |  |
| 1\|2^c^ | -4.16 | -4.55 to -3.78 | *<.001* | -4.20 | -4.59 – 3.82 | *<.001* |
| 2\|3^c^ | -1.63 | -1.82 to -1.43 | *<.001* | -1.64 | -1.83 – -1.44 | *<.001* |
| 3\|4^c^ | 1.30 | 1.11 – 1.48 | *<.001* | 1.32 | 1.14 – 1.50 | *<.001* |
| 4\|5^c^ | 1.64 | 1.45 – 1.84 | *<.001* | 1.67 | 1.48 – 1.86 | *<.001* |
| Duration of PSMS | 1.51 | 1.31 – 1.75 | *<.001* | 1.46 | 1.26 – 1.69 | *<.001* |
| Time between the start of the survey and the end of the most stressful time | 0.89 | 0.78 – 1.02 | .092 | 0.87 | 0.76 – 1.00 | .052 |
| MRRI | - | - | - | 0.86 | 0.70 – 1.07 | .185 |
| LF/HF | - | - | - | 0.86 | 0.74 – 1.00 | *.043* |
| VLF | - | - | - | 1.47 | 1.15 – 1.88 | *.002* |
| Tri-index | - | - | - | 1.04 | 0.83 – 1.30 | .748 |
| SDANN | - | - | - | 0.76 | 0.63 – 0.93 | *.007* |

^a^ RE: σ^2^ = 3.29, τ_00_ = 0.92 _participant_, ICC = 0.22,
Marginal R^2^ / Conditional R^2^ = 0.053 / 0.260. AIC = 3230.

^b^ RE: σ^2^ = 3.29, τ_00_ = 0.96 _participant_, ICC = 0.23,
Marginal R^2^ / Conditional R^2^ = 0.068 / 0.278. AIC = 3221.

^c^Estimates are reported for Threshold values instead of odds ratios.
