# Supplementary material for: Alignment Between Heart Rate Variability From Fitness Trackers and Perceived Stress: Perspectives From a Large-Scale In Situ Longitudinal Study of Information Workers
Source: JMIR Hum Factors. 2022 Aug 4;9(3):e33754. doi: 10.2196/33754 (PMC9389384; doi:10.2196/33754)
Supplement: Multimedia Appendix 5 [file humanfactors_v9i3e33754_app5.docx]

**Multimedia Appendix 5: Expanded versions of the tables included in the main paper including the threshold values for the ordinal models.**

Table S15. Expanded version of Table 7 including the threshold values. Model for perceived stress with VIF reduced HRV features derived from BBI data during normal work hours 8am to 6pm. Model fit on 14,695 Observations from 657 participants.

| Predictors | Perceived stress at the time of survey response (PSTR)^a^ | | | PSTR from anxiety, positive affect, and negative affect^b^ | | | PSTR from anxiety, positive affect, negative affect, and HRV^c^ | | |
| --- | --- | --- | --- | --- | --- | --- | --- | --- | --- |
|  | Estimate / OR | CI | *P* | Estimate / OR | CI | *P* | Estimate / OR | CI | *P* |
|  |  |  |  |  |  |  |  |  |  |
| 1\|2^d^ | -0.78 | -0.91 – -0.66 | *<.001* | -1.36 | -1.47 to -1.25 | *<.001* | -1.37 | -1.49 to -1.26 | *<.001* |
| 2\|3^d^ | 1.25 | 1.12 – 1.38 | .414 | 1.54 | 1.42 – 1.65 | *<.001* | 1.55 | 1.44 – 1.67 | *<.001* |
| 3\|4^d^ | 3.81 | 3.66 – 3.96 | *<.001* | 6.02 | 5.84 – 6.21 | *<.001* | 6.06 | 5.88 – 6.25 | *<.001* |
| 4\|5^d^ | 5.72 | 5.50 – 5.95 | *<.001* | 9.86 | 9.51 – 10.20 | *<.001* | 9.89 | 9.54 – 10.24 | *<.001* |
| MRRI | 0.95 | 0.89 – 1.02 | .160 | - | - | *-* | 1.01 | 0.94 – 1.09 | .753 |
| LF/HF | 0.86 | 0.82 – 0.91 | *<.001* | **-** | **-** | *-* | 0.85 | 0.81 – 0.90 | *<.001* |
| VLF | 1.54 | 1.42 – 1.67 | *<.001* | - | **-** | **-** | 1.31 | 1.20 – 1.43 | *<.001* |
| Tri-index | 0.88 | 0.83 – 0.94 | *<.001* | - | **-** | **-** | 0.94 | 0.88 – 1.01 | .099 |
| SDANN | 0.74 | 0.69 – 0.78 | *<.001* | - | **-** | *-* | 0.81 | 0.76 – 0.86 | *<.001* |
| Anxiety | - | - | *-* | 5.38 | 5.05 – 5.73 | *<.001* | 5.30 | 4.97 – 5.64 | *<.001* |
| Pos Aff. | - | - | *-* | 0.96 | 0.91 – 1.01 | *.*110 | 0.94 | 0.89 – 0.99 | *.011* |
| Neg Aff. | - | - | *-* | 2.52 | 2.37 – 2.68 | *<.001* | 2.53 | 2.38 – 2.69 | *<.001* |

^a^Random Effects: σ^2^ = 3.29, τ_00_ =2.25 _participant_, ICC = 0.41,
Marginal R^2^ / Conditional R^2^ = 0.022 / 0.420. AIC = 31602.

^b^Random Effects: σ^2^ = 3.29, τ_00_ =1.48 _participant_, ICC = 0.31,
Marginal R^2^ / Conditional R^2^ = 0.547 / 0.688. AIC =23709

^c^Random Effects: σ^2^ = 3.29, τ_00_ =1.52 _participant_, ICC = 0.32,
Marginal R^2^ / Conditional R^2^ = 0.548 / 0.691. AIC = 23561.

^d^Estimates are reported for Threshold values instead of odds ratios.

Table S16: Expanded version of Table 8 including the threshold values. Model for anxiety (CLMM) and negative affect (LMM) with VIF reduced HRV features derived from BBI data during normal work hours 8am to 6pm. Models fit on 14,695 Observations from 657 participants. P values lower than .05 are highlighted in italics.

| Predictors | Positive Affect^a^ | | | Negative Affect^b^ | | | Anxiety^c^ | | |
| --- | --- | --- | --- | --- | --- | --- | --- | --- | --- |
|  | Std. $\hat{\beta}$ | Std CI | *P* | IRR^d^ | CI | *P* | OR | CI | *P* |
|  |  |  |  |  |  |  |  |  |  |
| Intercept | -0.01 | -0.07 – 0.05 | *<.001* | 6.32 | 6.22 – 6.43 | <.001 | - | - | - |
| 1\|2^e^ | - | - | *-* | - | - | - | 0.05 | -0.09 – 0.18 | 0.496 |
| 2\|3^e^ | - | - | *-* | - | - | - | 2.44 | 2.30 – 2.58 | *<.001* |
| 3\|4^e^ | - | - | *-* | - | - | - | 4.39 | 4.22 – 4.56 | *<.001* |
| 4\|5^e^ | - | - | *-* | - | - | - | 6.03 | 5.79 – 6.27 | *<.001* |
| MRRI | -0.15 | -0.17 – -0.12 | *<.001* | 0.99 | -0.06 – -0.004 | .055 | 0.90 | 0.83 – 0.97 | *.004* |
| LF/HF | -0.08 | -0.10 – -0.07 | *<.001* | 1.00 | -0.03 – 0.01 | .877 | 0.92 | 0.87 – 0.97 | *.002* |
| VLF | 0.12 | 0.09 – 0.15 | *<.001* | 1.04 | 0.06 – 0.12 | *<.001* | 1.51 | 1.39 – 1.65 | *<.001* |
| Tri-index | 0.00 | -0.02 – 0.02 | *.911* | 0.98 | -0.08 – -0.03 | *.001* | 0.91 | 0.85 – 0.97 | *.005* |
| SDANN | -0.03 | -0.05 – -0.01 | *.002* | 0.98 | -0.07 – **-**0.03 | *<.001* | 0.76 | 0.72 – 0.81 | *<.001* |

^a^ Random Effects: σ^2^ = 9.03, τ_00_ =9.69 _participant_, ICC = 0.52,
Marginal R^2^ / Conditional R^2^ = 0.020 / 0.527.

^b^ Random Effects: σ^2^ = 0.15, τ_00_ =0.03 _participant_, ICC = 0.19,
Marginal R^2^ / Conditional R^2^ = 0.004 / 0.191.

^c^ Random Effects: σ^2^ = 3.29, τ_00_ =2.51 _participant_, ICC = 0.43,
Marginal R^2^ / Conditional R^2^ = 0.015 / 0.441.

^d^ Incidence Rate Ratios

^e^Estimates are reported for Threshold values instead of odds ratios.

Table S17: Expanded version of Table 9 including the threshold values. Prediction of PSTR, PSMS, and duration of PSMS with the same predictors -HRV during work hours - as in the best model in the main study. The models were fit with 1,373 observations from 327 participants.

| Predictors | PSTR^a^ | | | PSMS^b^ | | | Duration of PSMS^c^ | | |
| --- | --- | --- | --- | --- | --- | --- | --- | --- | --- |
|  | Est / OR | CI | *P* | Est / OR | CI | *P* | *Std* $\hat{\beta}$ | CI | *P* |
|  |  |  |  |  |  |  |  |  |  |
| Intercept |  |  |  |  |  |  | 0.02 | -0.06 – 0.10 | .643 |
| 1\|2^d^ | -2.16 | -2.38 – -1.94 | *<.001* | -4.14 | -4.52 – -3.75 | *<.001* | **-** | **-** | **-** |
| 2\|3^d^ | 0.18 | 0.01 – 0.35 | *.041* | -1.60 | -1.79 – -1.40 | *<.001* | **-** | **-** | **-** |
| 3\|4^d^ | 2.52 | 2.28 – 2.75 | *<.001* | 1.28 | 1.10 – 1.46 | *<.001* | **-** | **-** | **-** |
| 4\|5^d^ | 2.71 | 2.46 – 2.96 | *<.001* | 1.62 | 1.43 – 1.81 | *<.001* | **-** | **-** | **-** |
| MRRI | 0.98 | 0.79 – 1.23 | .889 | 0.86 | 0.70 – 1.07 | .184 | -0.03 | -0.12 – 0.07 | .585 |
| LF/HF | 0.84 | 0.73 – 0.98 | *.029* | 0.85 | 0.73 – 0.99 | *.036* | -0.02 | -0.09 – 0.05 | .569 |
| VLF | 1.56 | 1.22 – 1.99 | *<.001* | 1.54 | 1.21 – 1.97 | *<.001* | 0.15 | 0.05 – 0.25 | *.005* |
| Tri-index | 0.79 | 0.63 – 0.99 | *.039* | 0.99 | 0.79 – 1.24 | .932 | -0.11 | -0.19 – -0.03 | *.032* |
| SDANN | 0.75 | 0.61 – 0.91 | *.003* | 0.73 | 0.60 – 0.89 | *.002* | -0.10 | -0.20 – -0.01 | *.008* |

^a^ Random Effects: σ^2^ = 3.29, τ_00_ =1.21 _participant_, ICC = 0.27,
Marginal R^2^ / Conditional R^2^ = 0.032 / 0.292.

^b^ Random Effects: σ^2^ = 3.29, τ_00_ =0.97 _participant_, ICC = 0.22,
Marginal R^2^ / Conditional R^2^ = 0.023 / 0.245.

^c^ Random Effects: σ^2^ = 0.60, τ_00_ =0.40 _participant_, ICC = 0.40,
Marginal R^2^ / Conditional R^2^ = 0.019 / 0.414.

^d^Estimates are reported for Threshold values instead of odds ratios.
